# A hybrid recommendation algorithm based on user nearest neighbor model

**DOI:** 10.1038/s41598-024-66393-3

**Published:** 2024-07-25

**Authors:** Sheng Lv, Jiabin Wang, Fan Deng, Penggui Yan

**Affiliations:** school of engineering, Huaqiao University, 362011 Quanzhou, Fujian China

**Keywords:** Computer science, Scientific data

## Abstract

In the realm of e-commerce, personalized recommendations are a crucial component in enhancing user experience and optimizing sales efficiency. To address the inherent sparsity challenge prevalent in collaborative filtering algorithms within personalized recommendation systems, we propose a novel hybrid e-commerce recommendation algorithm based on the User-Nearest-Neighbor model. By integrating the user nearest neighbor model with other recommendation algorithms, this approach effectively mitigates data sparsity and facilitates a more nuanced understanding of the user-product relationship, consequently elevating recommendation quality and enhancing user experience. Taking into account considerations such as data scale and recommendation performance, we conducted experiments utilizing the Spark distributed platform. Empirical findings demonstrate the superiority of our hybrid algorithm over standalone collaborative filtering algorithms across various recommendation indicators.

## Introduction

The rapid expansion of the Internet has rendered e-commerce an indispensable facet of contemporary business operations. Within the e-commerce domain, recommender systems (RS)^1–5^ play a pivotal role in augmenting user satisfaction, boosting sales, and fortifying platform competitiveness^6^. Noteworthy is the substantial success RS has achieved across diverse business landscapes, exemplified by globally recognized platforms like Amazon, Netflix, TripAdvisor, and Last.fm^3^. However, in the era of big data, the development of high-caliber personalized e-commerce recommendation systems faces formidable hurdles, particularly in grappling with vast and sparsely populated user-item interaction datasets.

Collaborative filtering^7–10^ is a popular personalized recommendation technology that relies on the similarity between users or items to generate recommendations. In recent years, the optimization strategies for collaborative filtering models have been continuously enriched, as shown in the literature^11–13^. Within collaborative filtering models, matrix factorization (MF)^14^, multilayer perceptron (MLP)^15^, and neural collaborative filtering (NCF)^16^ are the most classic and widely used algorithms. However, these algorithms face significant challenges due to the high dimensionality and sparsity of the user-item interaction matrix^3,17^. In real-world scenarios, users typically interact with less than 1% of the total number of items. Consequently, the entire user-item interaction matrix is both high-dimensional and sparse. Due to this sparsity and high dimensionality, traditional collaborative filtering models struggle to effectively capture implicit user and item features, resulting in a decrease in recommendation quality.

To tackle the challenge posed by the sparsity of the user-item interaction matrix, we introduced an innovative hybrid recommendation algorithm^18^. The fundamental concept of this algorithm is to optimize the user-item interaction matrix and alleviate its data sparsity. The specific methodology is outlined as follows: initially, the missing values within the original user-item interaction matrix are forecasted using the user nearest neighbor model. Subsequently, the optimized user-item interaction matrix serves as the input for the collaborative filtering model. Ultimately, a personalized recommendation list is generated based on this processed data.

This method can effectively alleviate the sparsity of the original user-item interaction matrix and reduce the impact of matrix sparsity on the collaborative filtering model. Additionally, leveraging the distributed computing capabilities of the Apache Spark platform ensures efficient computation of high-dimensional and large-scale matrices.

## Definition of problems and related work

### Apache spark computing framework^19^

Apache Spark is an open-source distributed computing framework designed for processing and analyzing large-scale data. Unlike the traditional MapReduce computing method that relies on disk processing, Spark utilizes in-memory computing, allowing data to remain in memory. This memory-based capability significantly enhances Spark’s data processing speed^20^, especially for algorithms such as matrix factorization, as it enables iterative processing using data stored in memory, thereby accelerating the algorithm’s convergence.

Moreover, Spark introduces the concept of Resilient Distributed Datasets (RDDs), which are distributed, cacheable data structures that can share and reuse data between multiple computing tasks. This innovation not only enhances performance but also increases flexibility.

Spark is compatible with multiple programming languages, including Scala, Java, Python, and R, and it provides a wide range of libraries. These include Spark SQL^21^ for structured data processing, Spark Streaming^22^ for real-time data processing, machine learning libraries such as MLlib^23^ and ML, and the graph processing library Graph-X^24^. These libraries expedite the development of recommendation systems while also providing tools for data preprocessing, feature engineering, and model training.

### User-item interaction matrix

The User-Item Interaction Matrix is a widely utilized data structure in the field of personalized recommendation, employed to depict the interactions and associations between users and items. It is typically represented as a two-dimensional matrix, where rows correspond to users, columns correspond to items, and each element signifies the interaction between users and items. As illustrated in Table 1.

**Table 1:** User-Item Interaction Matrix.

User	Item				
	$Item_{1}$	...	$Item_{k}$	...	$Item_{n}$
$User_{1}$	$R_{1,1}$	...	$R_{1,k}$	...	$R_{1,n}$
...	...	...	...	...	...
$User_{j}$	$R_{j,1}$	...	$R_{j,k}$	...	$R_{j,n}$
...	...	...	...	...	...
$User_{m}$	$R_{m,1}$	...	$R_{m,k}$	...	$R_{m,n}$

## Algorithm design

### User nearest neighbor model

The basic principle of the User Nearest Neighbor (UNN) model is to make predictions based on the behavior of the users most similar to the target user. The core idea involves calculating the similarity between users based on the frequency of interactions with products and the set of interacted products. For each user u, the nearest neighbor user v is identified. Then, based on the interaction frequency of user v with some products that user u has not interacted with (but which the nearest neighbor user v has interacted with), and the similarity between users, the interaction frequency of user u with these non-interacted products is predicted. The specific calculation formula is as follows:

1 $$\begin{aligned} R_{u i}=\left\{ \begin{array}{cl}\max \left( {\text {Sim}}(u, v) \cdot R_{v i}, 1\right) , &{} i \notin I_{u} \text{ and } i \in I_{v} \\ R_{u i}, &{} i \in I_{u}\end{array}\right. \end{aligned}$$

Among them, $I_{u}$ and $I_{v}$ represent the sets of products interacted by users u and v respectively; *Sim*(*u*, *v*) represents the similarity between users u and v; $R_{ui}$ and $R_{vi}$ represent the interaction frequency of users u and v for product i respectively.

### Similarity metric

Traditional methods for calculating user similarity include Euclidean distance^25^, cosine similarity^1,26^, and Pearson correlation coefficient^17,26^. The specific formula is as follows:

- Euclidean distance
  2 $$\begin{aligned} Sim_{Euclidean}(u,v)=\frac{1}{\sqrt{ {\sum _{i\in I_{uv} }}(R_{ui} -R_{vi})^{2} } +1} \end{aligned}$$
- Cosine similarity
  3 $$\begin{aligned} Sim_{Cosine}(u,v)=\frac{ {\sum _{i\in I_{uv}}R_{ui}\cdot R_{vi}} }{\sqrt{ {\textstyle \sum _{i\in I_{uv} }}R_{ui}^{2} }\cdot \sqrt{ {\sum _{i\in I_{uv}} R_{vi}^2} } } \end{aligned}$$
- Pearson correlation coefficient
  4 $$\begin{aligned} Sim_{Pearson}(u,v)=\frac{ {\sum _{i\in I_{uv}}(R_{ui}-\bar{R_{u}}) \cdot (R_{vi}-\bar{R_{v}})} }{\sqrt{ {\sum _{i\in I_{uv}}(R_{ui}-\bar{R_{u}})^2} }\cdot \sqrt{{\sum _{i\in I_{uv}}(R_{vi}-\bar{R_{v}})^2} } } \end{aligned}$$

Where $I_{uv}$ represents the set of items co-interacted by both user u and v; $R_{ui}$ and $R_{vi}$ respectively indicate the interaction frequency of users *u* and *v* for item i; $\bar{R_{u}}$ and $\bar{R_{v}}$ represent the average interaction frequency of users *u* and *v* for their interacted items.

Traditional similarity measurement relies on the behavioral data of the items that users u and v interact with. However, due to the inherent sparsity of the user-item interaction matrix, the number of items that two users interact with is usually very limited, resulting in a large estimation error in the calculated user similarity. For example, if two users interact with only one item, but their interaction frequencies are the same, the traditional similarity measurement will consider them to have a high similarity, which is unreasonable.

To tackle this issue, our study introduces a weighted similarity measurement method. The core principles of this method are twofold: firstly, the higher the number of items two users interact with, the greater their similarity; secondly, within the set of interacted items, lower popularity indicates a stronger reflection of user differences. This article utilizes the Euclidean distance as an example to elucidate this concept. The specific calculation formula for weighted similarity is as follows:

5 $$\begin{aligned} pop_{i}= & {} \frac{\left| U_{i} \right| }{\left| U \right| } \cdot {\textstyle \sum _{u\in U_{i}}R_{ui}} \end{aligned}$$

6 $$\begin{aligned} Sim_{weight}(u,v)= & {} \frac{\left| I_{uv} \right| }{\sqrt{\left| I_{u} \right| \cdot \left| I_{v} \right| } }\cdot \frac{1}{\sqrt{ {\textstyle \sum _{i\in I_{uv}}\frac{1}{e^{pop_{i}}+1}\cdot (R_{ui}-R_{vi})^{2} } }+1 } \end{aligned}$$

Among them, $pop_{i}$ represents the popularity value of product i; U represents the set of all users; $U_{i}$ represents the set of users who have interacted with product i; $R_{ui}$ represents the interaction frequency of user u with product i.

By considering both the number of co-interactions and item popularity weights, this weighted similarity measurement method minimizes estimation errors and offers a more precise depiction of user similarity.

### Alternating least squares method

Alternating Least Squares (ALS)^27^ is a widely used MF optimization algorithm. Taking implicit feedback^28,29^ data as an example, the specific steps of the ALS algorithm are summarized as follows:

- Randomly initialize the user matrix *P* and the item matrix *Q*.
  7 $$\begin{aligned} P= & {} (p_{1},p_{2},...,p_{n}) \end{aligned}$$
  8 $$\begin{aligned} Q= & {} (q_{1},q_{2},...,q_{m}) \end{aligned}$$
- Treat the user matrix *P* as a fixed value and update the item matrix *Q*.
  9 $$\begin{aligned} q_{i}=(\sum _{u\in U}C_{ui}\cdot p_{u}\cdot p_{u}^{T}+\lambda \cdot E )^{-1}\cdot \sum _{u\in U} C_{ui}\cdot y_{ui}\cdot p_{u} \end{aligned}$$
- Treat the item matrix *Q* as a fixed value and update the user matrix *P*.
  10 $$\begin{aligned} p_{u}=(\sum _{i\in I}C_{ui}\cdot q_{i}\cdot q_{i}^{T}+\lambda \cdot E )^{-1}\cdot \sum _{i\in I} C_{ui}\cdot y_{ui}\cdot q_{i} \end{aligned}$$
- Continue alternating iterations until the loss function converges or reaches the maximum iteration limit.
  11 $$\begin{aligned} Loss(P,Q)=\sum _{u\in U} \sum _{i\in I}C_{ui}\cdot (y_{ui}-p_{u}^{T}\cdot q_{i} )^2+\lambda \cdot (\sum _{u\in U}\left\| p_{u} \right\| ^2+\sum _{i\in I}\left\| q_{i} \right\| ^2 ) \end{aligned}$$

Where n and m represent the number of users and items respectively; $p_{u}$ and $q_{i}$denote the feature vectors of user *u* and item *i* respectively; *U* and *I* represent the sets of users and items respectively; $C_{ui}$ represents the confidence of user *u* for item *i*; $y_{ui}$ indicates whether user *u* prefers item *i*; $\lambda$ denotes the regularization coefficient; *E* represents the identity matrix. The specific formulas for $C_{ui}$ and $y_{ui}$ are as follows:

12 $$\begin{aligned} C_{ui}= & {} 1+\alpha \cdot R_{ui} \end{aligned}$$

13 $$\begin{aligned} y_{ui}= & {} {\left\{ \begin{array}{ll} 1 &{} \text {, } R_{ui}>0 \\ 0 &{} \text {, } R_{ui}=0\end{array}\right. } \end{aligned}$$

Where $\alpha$ represents the confidence coefficient; $R_{ui}$ represents the interaction frequency of user u for product i.

### Multilayer perceptron

Multilayer Perceptron (MLP)^15^ is a foundational feedforward artificial neural network, constituting a fundamental component of deep learning architectures. It typically comprises an embedding layer, one or more hidden layers, and an output layer. The training process of the MLP model unfolds as follows:

- *Embedding layer*: Map users and products to a low-dimensional vector space through an embedding matrix.
  14 $$\begin{aligned} p_{u}= & {} P_{e}\cdot u_{One-Hot} \end{aligned}$$
  15 $$\begin{aligned} q_{i}= & {} Q_{e}\cdot i_{One-Hot} \end{aligned}$$
  Among them, $p_{u}$ and $q_{i}$ represent the low-dimensional feature vectors of user u and product i respectively; $P_{e}$ and $Q_{e}$ both represent embedding matrices; $u_{One-Hot}$ and $i_{One-Hot}$ represent the one-hot encoding of user u and product i respectively.
- *Hidden layer*: Connect the embedded vectors into a long vector, and learn nonlinear feature interactions through multi-layer fully connected networks.
  16 $$\begin{aligned} h_{1}= & {} \phi (W_{1}[p_{u}||q_{i}]+b_{1}) \end{aligned}$$
  17 $$\begin{aligned} h_{l}= & {} \phi (W_{l}h_{l-1} +b_{l}),l=2,3,...,L \end{aligned}$$
  Among them, $\phi$ represents the hidden layer activation function; || represents the connection operation; W and b represent the weight and bias of the hidden layer respectively; L represents the number of hidden layers.
- *Output layer*: Predict the user’s rating or preference for the product.
  18 $$\begin{aligned} \hat{y}_{ui}=\sigma (W_{out}h_{L}+b_{out}) \end{aligned}$$
  Where $\sigma$ represents the output layer activation function; $W_{out}$ and $b_{out}$ represent the weight and bias of the output layer respectively.

### Neural collaborative filtering

**Figure 1:**
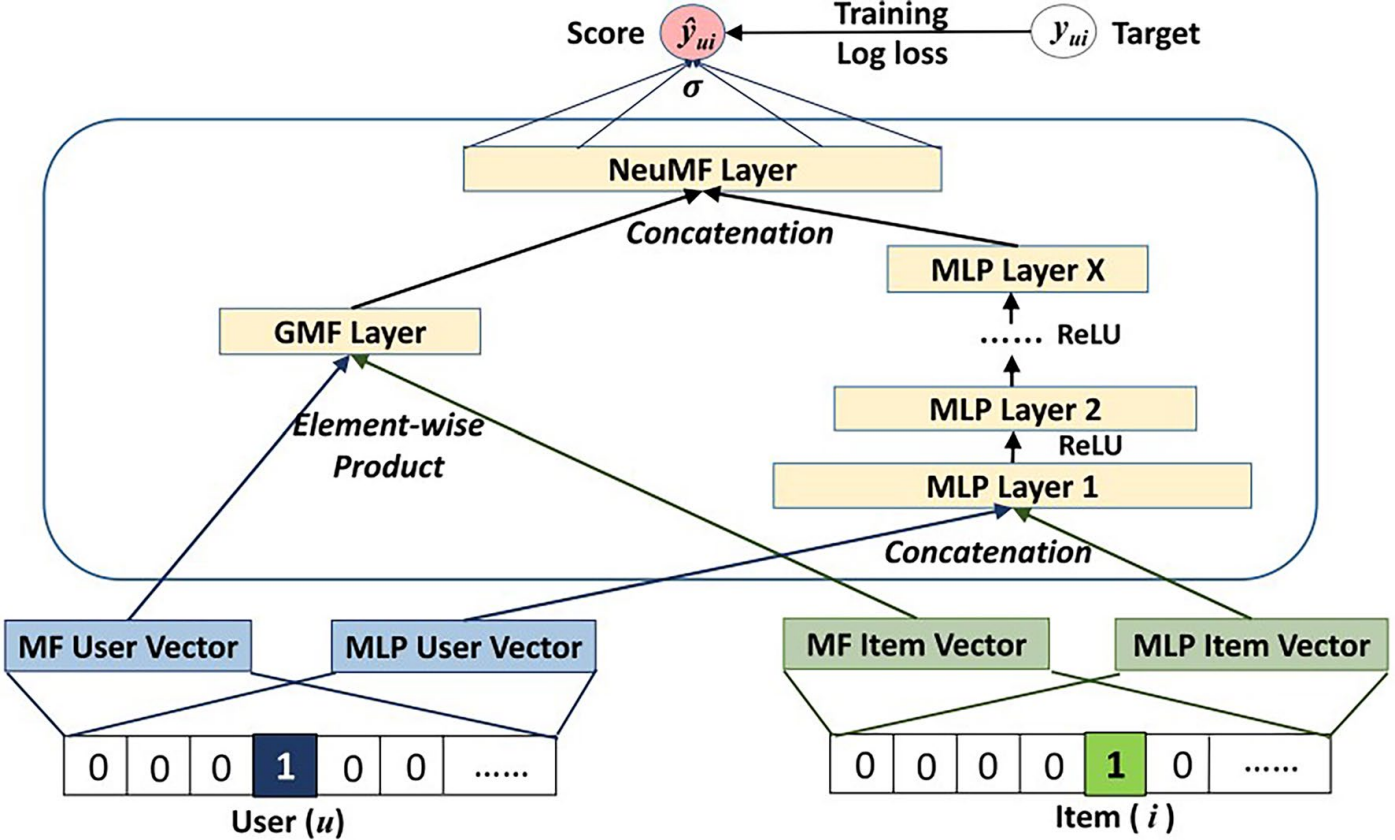
Schematic diagram of the NCF model framework^16^.

Neural collaborative filtering (NCF)^16^ is a recommendation algorithm that integrates deep learning with collaborative filtering principles. It leverages neural networks to capture the nonlinear relationship between users and products. The NCF model combines the strengths of MLP and generalized matrix factorization (GMF), allowing it to achieve superior recommendation outcomes in high-dimensional sparse data scenarios. The specific training methodology of the NCF model is depicted in Fig. 1:

- *Embedding layer*: Map users and products to low-dimensional vector space through the embedding matrix.
  19 $$\begin{aligned} p_{u}^{G}= & {} P_{e}^{G} \cdot u_{One-Hot} \end{aligned}$$
  20 $$\begin{aligned} q_{i}^{G}= & {} Q_{e}^{G} \cdot i_{One-Hot} \end{aligned}$$
  21 $$\begin{aligned} p_{u}^{M}= & {} P_{e}^{M} \cdot u_{One-Hot} \end{aligned}$$
  22 $$\begin{aligned} q_{i}^{M}= & {} Q_{e}^{M} \cdot i_{One-Hot} \end{aligned}$$
  Among them, $p_{u}^{G}$ and $q_{i}^{G}$ represent the low-dimensional feature vectors of user u and item i based on the GMF model respectively; $P_{e}^{G}$ and $Q_{e}^{G}$ both represent the embedding matrix based on the GMF model; $p_{u}^{M}$ and $q_{i}^{M}$ represent the low-dimensional feature vectors of user u and item i based on the MLP model respectively; $P_{e}^{M}$ and $Q_{e}^{M}$ both represent the embedding matrix based on the MLP model; $u_{One-Hot}$ and $i_{One-Hot}$ represent the one-hot encoding of user u and item i respectively.
- *Hidden layer*: Captures the complex interactive relationship between users and items through multiple nonlinear transformations and feature extraction.
  23 $$\begin{aligned} h_{GMF}= & {} p_{u}^{G}\odot q_{i}^{G} \end{aligned}$$
  24 $$\begin{aligned} h_{MLP}= & {} \phi _{L}(W_{L}(\phi _{L-1}(...\phi _{1}(W_{1}[p_{u}^{M}|| q_{i}^{M}]+b_{1})...)+b_{L-1})+b_{L}) \end{aligned}$$
  25 $$\begin{aligned} h_{concat}= & {} [h_{GMF}||h_{MLP}] \end{aligned}$$
  Among them, $\odot$ represents element-by-element multiplication; $h_{GMF}$ and $h_{MLP}$ represent the hidden layer embedding vectors obtained by the GMF model and the MLP model respectively; $\phi _{L}$ represents the activation function of the Lth layer of the hidden layer of the MLP model; $W_{L}$ and $b_{L}$ represent the weight and bias of the Lth layer of the hidden layer of the MLP model respectively; $h_{concat}$ represents the embedded vector after concatenation; || represents the concatenation operation.
- *Output layer*: Predict the user’s evaluation or preference for the product.
  26 $$\begin{aligned} \hat{y}_{ui}=\sigma (W_{out}h_{concat}+b_{out}) \end{aligned}$$
  Among them, $\sigma$ represents the output layer activation function; $W_{out}$ and $b_{out}$ represent the weight and bias of the output layer respectively.

The ALS algorithm treats all non-interacted products as negative examples during training. However, in reality, due to data sparsity, there’s a significant imbalance between positive and negative examples. Even with negative sampling, there can still be a substantial number of false negative examples. Additionally, while the MLP algorithm and NCF algorithm excel in capturing the nonlinear relationships between users and products, this strength can lead to overfitting issues when dealing with sparse matrices. Therefore, optimizing the user-product interaction matrix becomes crucial in mitigating these challenges.

This paper integrates the UNN model with the ALS, MLP, and NCF algorithms, respectively. Optimizing the user-item interaction matrix, addresses the limitations of individual recommendation algorithms in dealing with data sparsity, thereby enhancing recommendation quality.

## Experimental analysis

This chapter leverages the Spark platform to evaluate the recommendation quality of the hybrid e-commerce recommendation algorithm based on the UNN model proposed in this paper. Through experiments, its performance is compared with various collaborative filtering algorithms. The results demonstrate that the hybrid recommendation algorithm outperforms single algorithms across multiple indicators.

### Data set

Dataset 1: This dataset is sourced from the Alibaba mobile e-commerce platform and provided by Alibaba Cloud Tian-chi Laboratory. It comprises 834 users and 350,889 distinct items, with 1,048,575 records of user-item interactions, resulting in a data sparsity of 99.865%. The interactions between users and items in this dataset include browsing, bookmarking, adding to cart, and purchasing. Dataset 1 link: Dataset 1 link.

Dataset 2: This dataset is derived from a Taobao user behavior dataset provided by Alibaba, designed for research on implicit feedback recommendation problems. It includes 987,994 users and 4,162,024 distinct items, with 100,150,807 records of user-item interactions. For this experiment, a subset of the data was selected, comprising 1,000 users and 48,488 distinct items, with 73,980 records of user-item interactions, resulting in a data sparsity of 99.887%. The interactions between users and items in this dataset include browsing, bookmarking, adding to cart, and purchasing. Dataset 2 link: Dataset 2 link.

Dataset 3: This dataset is sourced from the Kaggle website. Due to the presence of many users interacting with only a few items in this dataset, to appropriately partition the training and testing sets, this study extracted a subset of valid data, comprising 7,023 users and 40,022 unique items, with 191,711 user-item interaction records. The data sparsity is 99.965%. The interactions between users and items in this dataset include browsing, adding to cart, and purchasing. Dataset 3 link: Dataset 3 link.

### Metric

This article employs classic indicators such as F1 score, Hit Rate (HR), and Normalized Discounted Cumulative Gain (NDCG) as evaluation criteria^30^. The specific calculation formulas are as follows:

- F1 score
  27 $$\begin{aligned} P= & {} \frac{1}{\left| U \right| } \cdot {\textstyle \sum _{u\in U} \frac{\left| R(u)\cap T(u) \right| }{\left| R(u) \right| } } \end{aligned}$$
  28 $$\begin{aligned} R= & {} \frac{1}{\left| U \right| } \cdot {\textstyle \sum _{u\in U} \frac{\left| R(u)\cap T(u) \right| }{\left| T(u) \right| } } \end{aligned}$$
  29 $$\begin{aligned} F1= & {} \frac{2\times P\times R}{P+R} \end{aligned}$$
  Among them, *P* and *R* represent the precision rate and recall rate respectively. *U* represents the set of users, *R*(*u*) represents the recommendation list of user u, and *T*(*u*) represents the actual purchase list of user *u*.
- Hit Rate
  30 $$\begin{aligned} HR=\frac{1}{\left| U \right| } \cdot {\textstyle \sum _{u\in U}hits(u)} \end{aligned}$$
  Among them, *U* represents the set of users; *hits*(*u*) represents whether the product purchased by user *u* appears in the recommendation list. If it does, it is represented as 1; otherwise, it is represented as 0.
- Normalized Discounted Cumulative Gain
  31 $$\begin{aligned} DCG_{n}= & {} {\textstyle \sum _{n=1}^{\left| R(u) \right| }\frac{1}{\log _{2}{(rel_{n} +1)} } } \end{aligned}$$
  32 $$\begin{aligned} IDCG_{n}= & {} {\textstyle \sum _{n=1}^{\left| R(u)\cap T(u) \right| }\frac{1}{\log _{2}{(rel_{n} +1)} } } \end{aligned}$$
  33 $$\begin{aligned} NDCG= & {} \frac{1}{\left| U \right| } \cdot {\textstyle \sum _{u\in U}\frac{DCG_{n}}{IDCG_{n}} } \end{aligned}$$
  Among them, $DCG_{u}$ and $IDCG_{u}$ respectively represent the discounted cumulative gain and ideal cumulative gain of the user’s recommended list. *R*(*u*) and *T*(*u*) represent the user’s recommended list and actual purchase list respectively. $rel_{n}$ represents whether the user will purchase the nth product in the recommended list. If they will, it is n; otherwise, it is 0. U represents the set of users.

### The general framework of the experiment

The hybrid recommendation algorithm proposed in this paper, based on the UNN recommendation and MF, mainly consists of two parts: the UNN model and the MF model.

**Figure 2:**
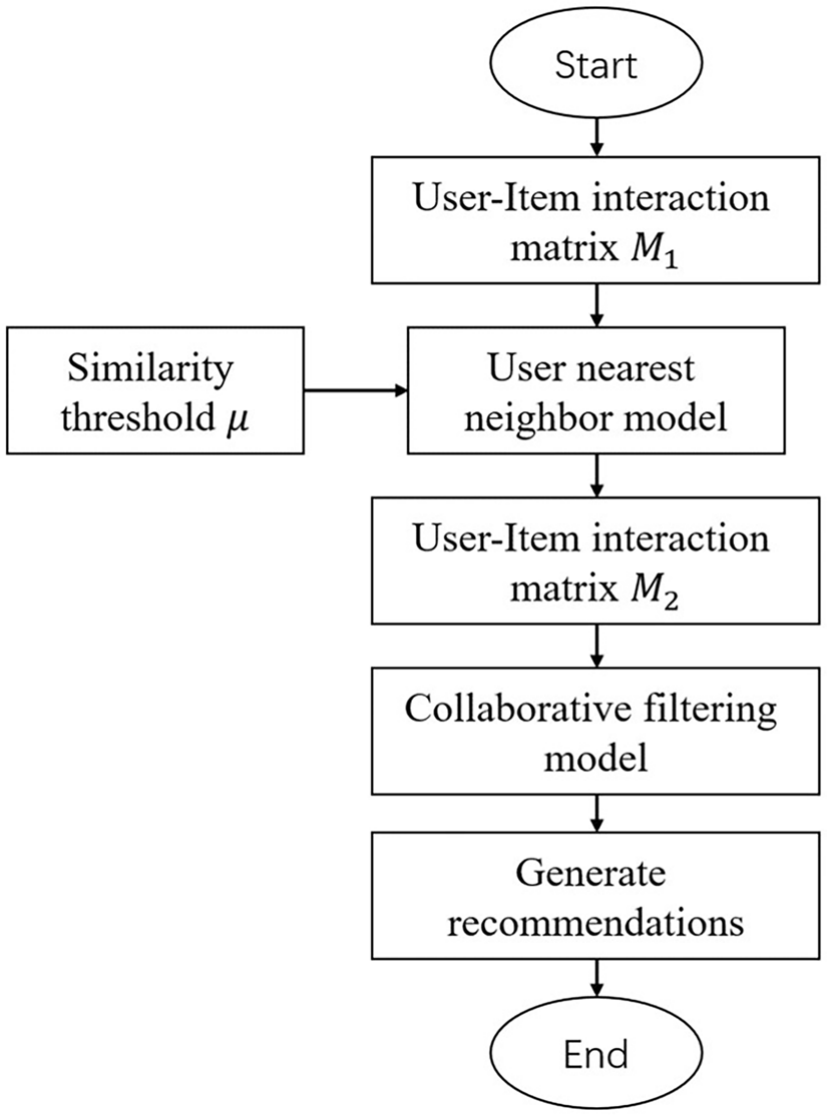
General framework diagram of the algorithm.

First, the user-item interaction matrix $M_{1}$ is optimized through the UNN model to predict the user’s preferences for items that have not been interacted with before, thereby generating a denser user-item interaction matrix $M_{2}$. Then, the collaborative filtering model is trained on the optimized user-item interaction matrix $M_{2}$ to effectively mitigate the negative impact of matrix sparsity on the collaborative filtering model. Finally, recommendations are generated based on the potential feature vectors of users and items. The specific workflow is illustrated in Fig. 2.

The similarity threshold $\mu$ serves as an independent variable in the UNN model, controlling the prediction accuracy within the model. Generally, the quality of the user-item interaction matrix $M_{2}$ increases as the similarity threshold $\mu$ decreases, reaching a peak before gradually declining.

### Experimental results and analysis

Next, we will conduct experiments using three datasets on the Spark platform to verify the recommendation quality of our proposed hybrid recommendation algorithm based on the UNN model under different similarity thresholds and compare it with other collaborative filtering recommendation algorithms.

In the ALS algorithm, the dimension of the potential feature vector is set to 32 to 80. In the MLP algorithm, the number of neurons in the embedding layer ranges from 32 to 80, the number of hidden layers is 2, the number of neurons is 32 and 64 respectively, and the number of neurons in the output layer is 100. In the NCF algorithm, the embedding dimension is set to 32 to 80, and the neural network structure is the same as the MLP algorithm.

**Figure 3:**
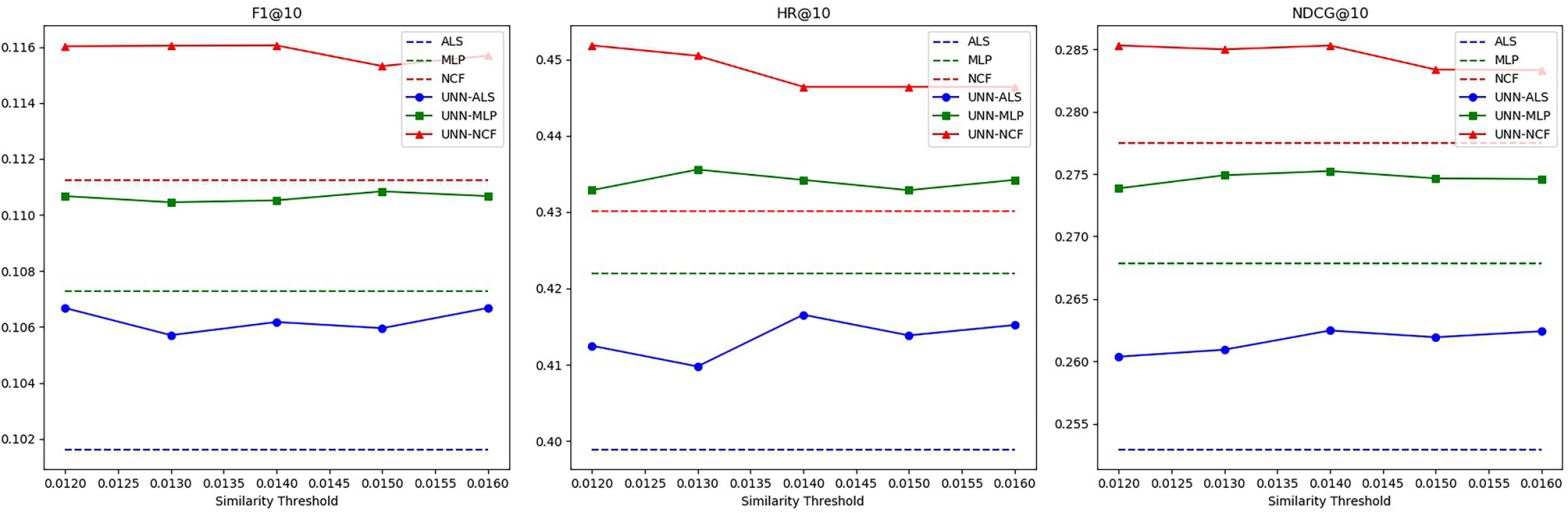
Schematic diagram of the effectiveness of the UNN model based on different similarity thresholds in Dataset 1.

**Figure 4:**
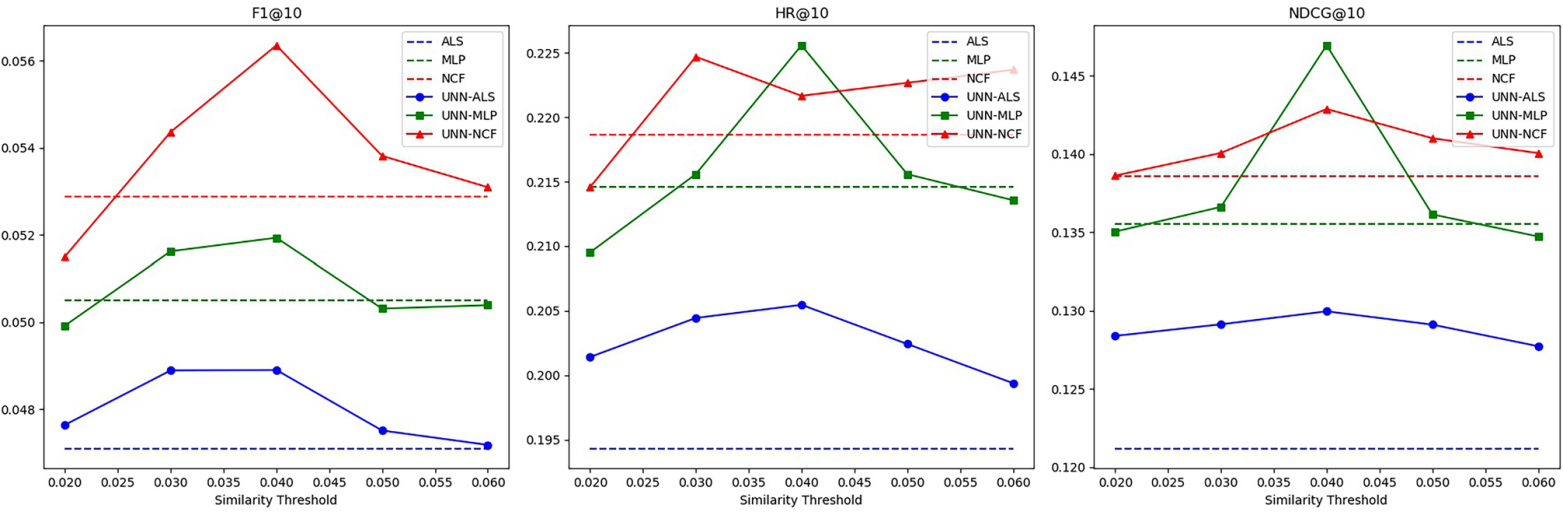
Schematic diagram of the effectiveness of the UNN model based on different similarity thresholds in Dataset 2.

**Figure 5:**
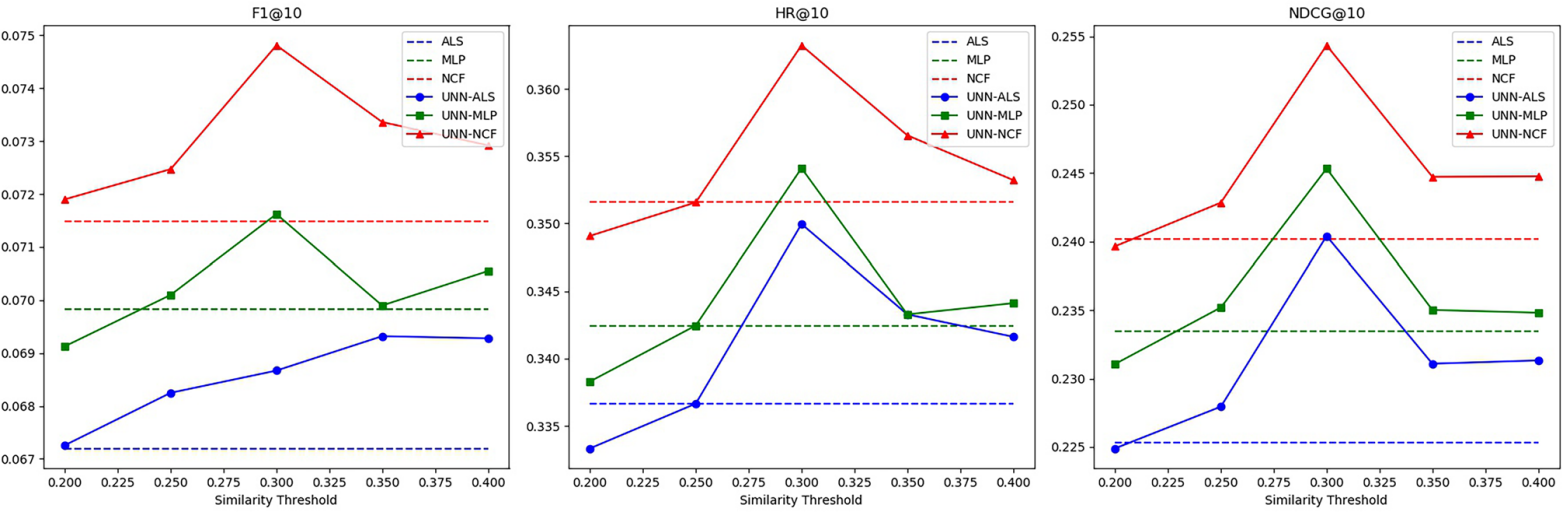
Schematic diagram of the effectiveness of the UNN model based on different similarity thresholds in Dataset 3.

In the first part of the experiment, we will discuss in detail the effectiveness of the UNN model under different similarity thresholds. We will select an optimal similarity threshold for each dataset. Figures 3, 4, 5 and Tables 2, 3, 4 present some experimental data.

**Table 2:** Effectiveness of UNN models based on different similarity thresholds in Dataset 1.

Algorithm	Similarity threshold	Metrics		
		F1@10	HR@10	NDCG@10
ALS		0.10163	0.39891	0.25296
MLP		0.10727	0.42198	0.26784
NCF		0.11123	0.43012	0.27751
ALS+UNN	0.013	0.10571	0.40977	0.26094
	**0.014**	**0.10618**	**0.41655**	**0.26248**
	0.015	0.10596	0.41384	0.26194
MLP+UNN	0.013	0.11045	0.43555	0.27490
	**0.014**	**0.11052**	**0.43419**	**0.27524**
	0.015	0.11084	0.43284	0.27465
NCF+UNN	0.013	0.11605	0.45047	0.28499
	**0.014**	**0.11606**	**0.44640**	**0.28530**
	0.015	0.11532	0.44640	0.28337

Significant values are in bold.

**Table 3:** Effectiveness of UNN models based on different similarity thresholds in Dataset 2.

Algorithm	Similarity threshold	Metrics		
		F1@10	HR@10	NDC G@10
ALS		0.04710	0.19433	0.12120
MLP		0.05049	0.21457	0.13552
NCF		0.05287	0.21862	0.13858
ALS+UNN	0.03	0.04889	0.20445	0.12913
	**0.04**	**0.04890**	**0.20547**	**0.12996**
	0.05	0.04751	0.20243	0.12911
MLP+UNN	0.03	0.05162	0.21558	0.13661
	**0.04**	**0.05193**	**0.22558**	**0.14694**
	0.05	0.05031	0.21558	0.13613
NCF+UNN	0.03	0.05436	0.22469	0.14006
	**0.04**	**0.05635**	**0.22166**	**0.14287**
	0.05	0.05381	0.22267	0.14100

Significant values are in bold.

**Table 4:** Effectiveness of UNN models based on different similarity thresholds in Dataset 3.

Algorithm	Similarity threshold	Metrics		
		F1@10	HR@10	NDCG@10
ALS		0.06720	0.33665	0.22530
MLP		0.06983	0.34245	0.23347
NCF		0.07149	0.35157	0.24017
ALS+UNN	0.25	0.06825	0.33665	0.22795
	**0.3**	**0.06867**	**0.34996**	**0.24040**
	0.35	0.06932	0.34328	0.23109
MLP+UNN	0.25	0.07010	0.34245	0.23520
	**0.3**	**0.07162**	**0.35411**	**0.24533**
	0.35	0.06990	0.34328	0.23502
NCF+UNN	0.25	0.07247	0.35157	0.24284
	**0.3**	**0.07481**	**0.36323**	**0.25434**
	0.35	0.07336	0.35655	0.24473

Significant values are in bold.

As observed from the above charts, the hybrid recommendation algorithm we proposed exhibits significant sensitivity to changes in the similarity threshold. The characteristics of this sensitivity are as follows:

When the similarity threshold is set low, the UNN model generates a large amount of predicted interaction data. This data can be utilized as filler data to enrich the user-item interaction matrix and alleviate its sparsity. However, due to the less accurate prediction data generated by the UNN model at this similarity threshold, the negative impact of reduced data accuracy surpasses the positive impact of increased data density. Consequently, the entire algorithm performs poorly on some indicators.

As the similarity threshold increases, the amount of predicted interaction data generated by the UNN model decreases, while the accuracy increases. At this stage, the positive impact of increased data accuracy outweighs the negative impact of reduced data density. Therefore, all indicators of the entire algorithm show an upward trend.

When the similarity threshold reaches a certain value, although the amount of predicted interaction data generated by the UNN model will further decrease and the accuracy of predicted interaction data will increase, it essentially reaches a balanced state. Here, the positive impact of the latter and the negative impact of the former offset each other. Consequently, the recommendation quality of the entire algorithm continues to fluctuate. Eventually, as the amount of predicted interaction data generated by the UNN model approaches zero, the performance of the hybrid algorithm across various indicators will converge to that of a single algorithm.

Table 5 gives the basic information of the user-product interaction matrix based on these three datasets at different similarity thresholds. Among them, DS represents data sparsity, FV represents the amount of data filled in the user-item interaction matrix by the UNN model, and FVR represents the percentage of FV in the original data volume.

**Table 5:** Basic information table of user-item interaction matrix under different similarity thresholds.

Data set	Properties	Similarity threshold				
Data set 1		0.01	0.012	0.014	0.016	0.018
	DS	99.85%	99.86%	99.86%	99.86%	99.86%
	FV	35529	23952	12654	6811	3770
	FVR	8.94%	6.03%	3.19%	1.72%	0.95%
Data set 2		0.02	0.03	0.04	0.05	0.06
	DS	99.83%	99.86%	99.87%	99.88%	99.88%
	FV	24353	13257	7231	3794	2256
	FVR	46.50%	25.26%	13.78%	7.23%	4.30%
Data set 3		0.2	0.25	0.3	0.35	0.4
	DS	99.96%	99.96%	99.96%	99.96%	99.96%
	FV	10459	6027	3478	1988	1045
	FVR	12.56%	7.24%	4.18%	2.38%	1.26%

**Figure 6:**
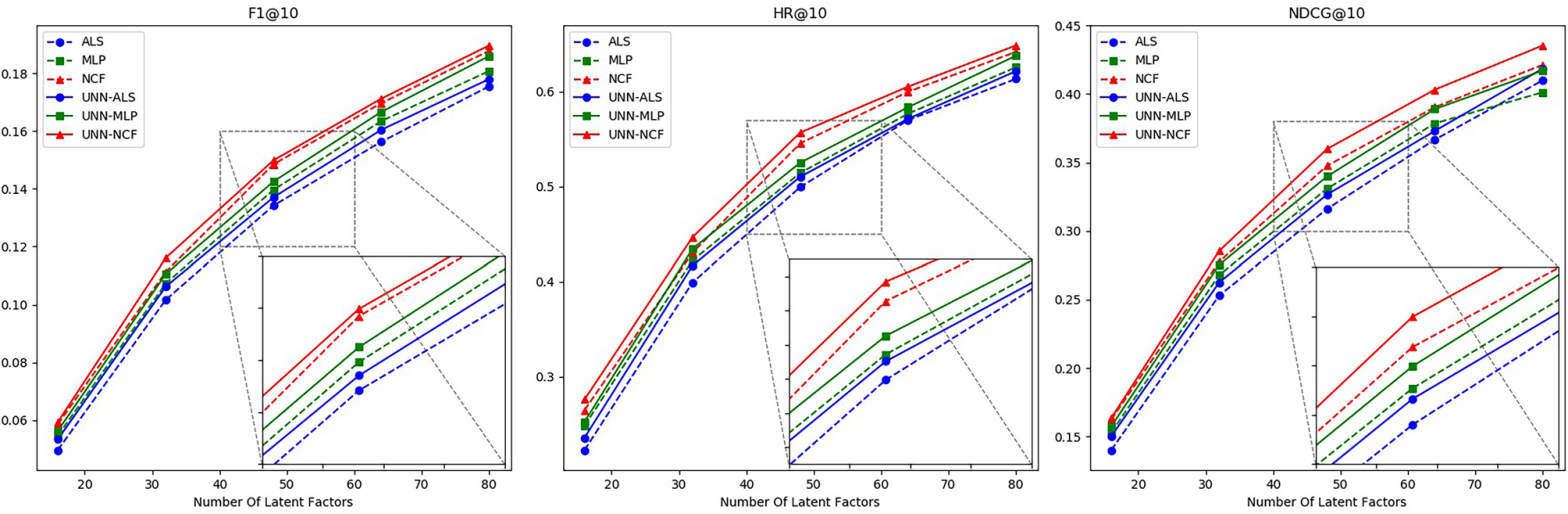
Schematic diagram of the effectiveness of the UNN model based on different latent factors in Dataset 1.

**Figure 7:**
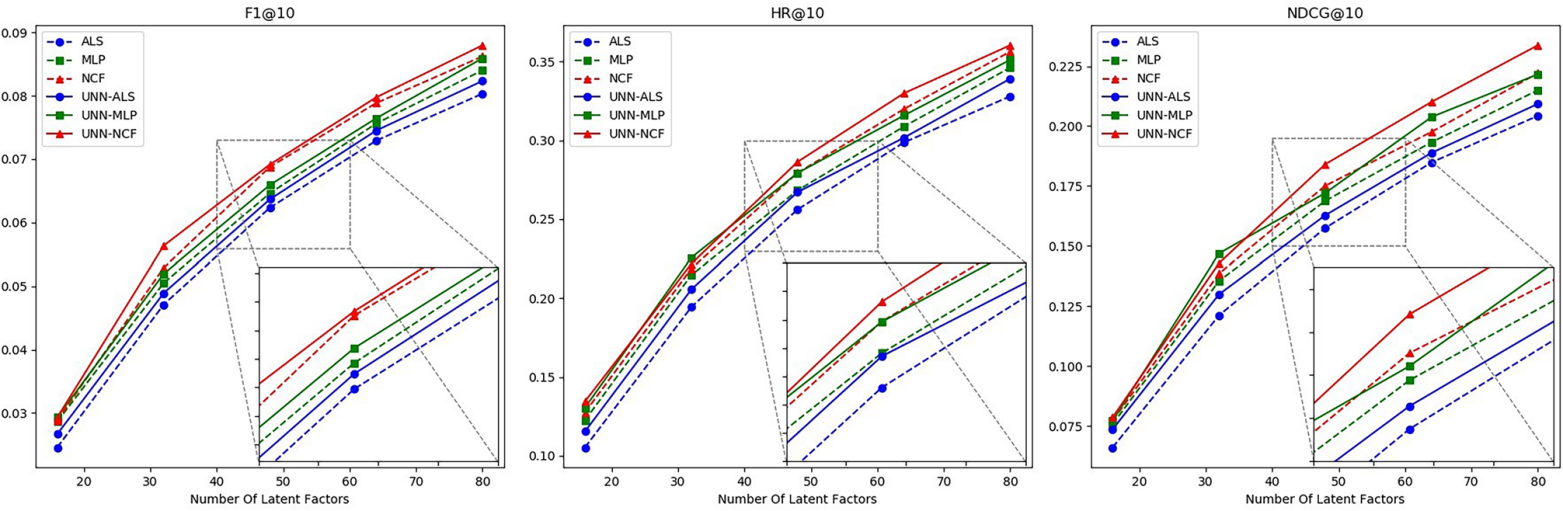
Schematic diagram of the effectiveness of the UNN model based on different latent factors in Dataset 2.

**Figure 8:**
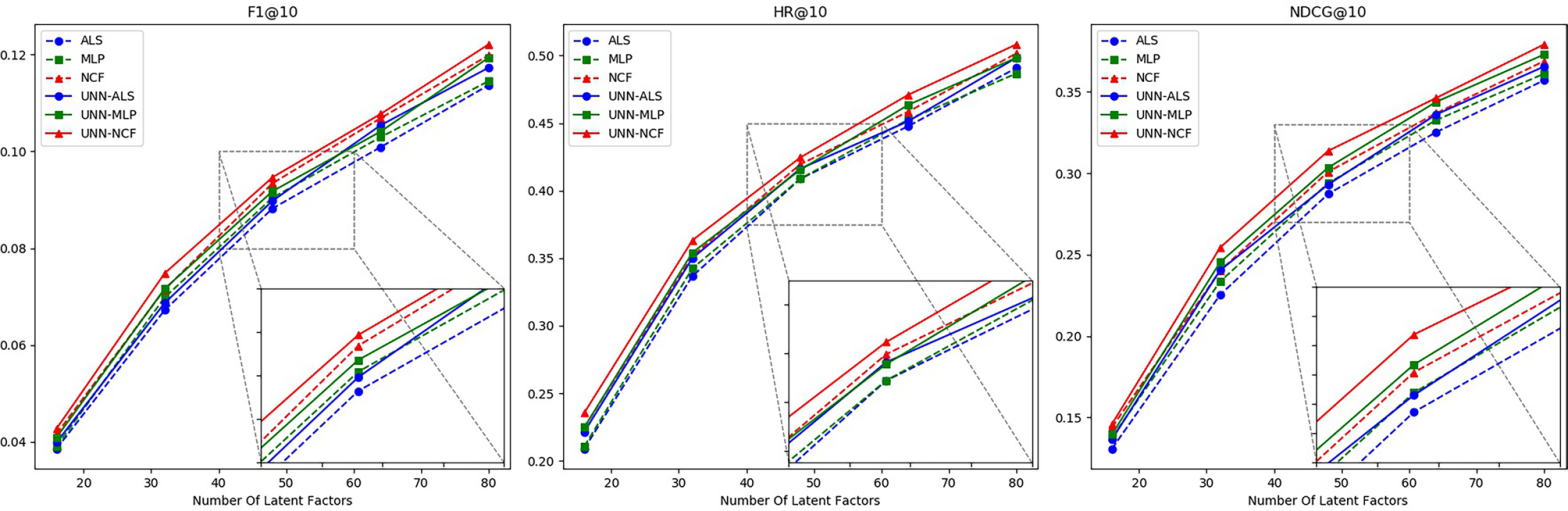
Schematic diagram of the effectiveness of the UNN model based on different latent factors in Dataset 3.

In the second part of the experiment, building upon the results from the discussion on the similarity threshold, we delve deeper into the recommendation quality of the hybrid algorithm and the single algorithm under varying implicit feature vector dimensions. This aims to validate the stability of the hybrid algorithm. The specific details are presented in Figs. 6, 7, 8, while detailed experimental data are provided in Tables 6, 7, 8.

**Table 6:** Effectiveness of UNN models based on different latent factors in Dataset 1.

Algorithm	Metrics	Number of latent factors				
		16	32	48	64	80
ALS	F1	0.04966	0.10163	0.13417	0.15628	0.17545
	HR	0.22252	0.39891	0.49932	0.56988	0.61330
	NDCG	0.14009	0.25296	0.31596	0.36655	0.40985
MLP	F1	0.05466	0.10727	0.13962	0.16343	0.18076
	HR	0.24830	0.42198	0.51424	0.57666	0.62550
	NDCG	0.15296	0.26784	0.33074	0.37846	0.40119
NCF	F1	0.05861	0.11123	0.14846	0.16964	0.18773
	HR	0.26458	0.43012	0.54545	0.59972	0.64179
	NDCG	0.16240	0.27751	0.34748	0.39026	0.42120
ALS+UNN	F1	0.05338	0.10618	0.13704	0.16041	0.17797
	HR	0.23559	0.41655	0.51018	0.57123	0.62144
	NDCG	0.15030	0.26248	0.32645	0.37293	0.41842
MLP+UNN	F1	0.05632	0.11052	0.14250	0.16663	0.18593
	HR	0.25237	0.43419	0.52510	0.58344	0.63772
	NDCG	0.15754	0.27524	0.33970	0.38891	0.41697
NCF+UNN	F1	0.05938	0.11606	0.14985	0.17120	0.18960
	HR	0.27594	0.44640	0.55681	0.60515	0.64857
	NDCG	0.16359	0.28530	0.35978	0.40310	0.43536

**Table 7:** Effectiveness of UNN models based on different latent factors in Dataset 2.

Algorithm	Metrics	Number of latent factors				
		16	32	48	64	80
ALS	F1	0.02457	0.04710	0.06234	0.07292	0.08033
	HR	0.10526	0.19433	0.25607	0.29858	0.32793
	NDCG	0.06611	0.12120	0.15753	0.18487	0.20431
MLP	F1	0.02866	0.05049	0.06462	0.07562	0.08403
	HR	0.12246	0.21457	0.26821	0.30870	0.34615
	NDCG	0.07584	0.13552	0.16883	0.19330	0.21499
NCF	F1	0.02885	0.05287	0.06878	0.07884	0.08622
	HR	0.12753	0.21862	0.27935	0.31983	0.35627
	NDCG	0.07729	0.13858	0.17512	0.19761	0.22208
ALS+UNN	F1	0.02667	0.04890	0.06367	0.07451	0.08234
	HR	0.11538	0.20547	0.26709	0.30162	0.33907
	NDCG	0.07369	0.12996	0.16283	0.18887	0.20928
MLP+UNN	F1	0.02936	0.05193	0.06593	0.07645	0.08591
	HR	0.13045	0.22558	0.27923	0.31573	0.35109
	NDCG	0.07725	0.14694	0.17210	0.20381	0.22162
NCF+UNN	F1	0.02929	0.05635	0.06916	0.07975	0.08797
	HR	0.13449	0.22166	0.28632	0.32983	0.36032
	NDCG	0.07869	0.14287	0.18412	0.21011	0.23373

**Table 8:** Effectiveness of UNN models based on different latent factors in Dataset 3.

Algorithm	Metrics	Number of latent factors				
		16	32	48	64	80
ALS	F1	0.03860	0.06720	0.08823	0.10088	0.11368
	HR	0.20896	0.33665	0.40878	0.44776	0.49087
	NDCG	0.13090	0.22530	0.28724	0.32520	0.35709
MLP	F1	0.03907	0.06983	0.09044	0.10295	0.11453
	HR	0.21061	0.34245	0.40879	0.45274	0.48673
	NDCG	0.13781	0.23347	0.29387	0.33259	0.36110
NCF	F1	0.04164	0.07149	0.09342	0.10686	0.11985
	HR	0.22471	0.35157	0.41956	0.45854	0.50165
	NDCG	0.14399	0.24017	0.30081	0.33681	0.36886
ALS+UNN	F1	0.03989	0.06867	0.08981	0.10541	0.11730
	HR	0.22144	0.34996	0.41630	0.45144	0.49844
	NDCG	0.13673	0.24040	0.29302	0.33606	0.36550
MLP+UNN	F1	0.04095	0.07162	0.09179	0.10410	0.11937
	HR	0.22476	0.35411	0.41547	0.46357	0.49839
	NDCG	0.13975	0.24533	0.30344	0.34377	0.37290
NCF+UNN	F1	0.04276	0.07481	0.09466	0.10773	0.12211
	HR	0.23554	0.36323	0.42459	0.47102	0.50834
	NDCG	0.14605	0.25434	0.31369	0.34621	0.37913

As observed from the above chart, by selecting an appropriate similarity threshold, the UNN model enhances the data density of the user-item interaction matrix while preserving its accuracy to the greatest extent. Consequently, the hybrid algorithm combined with UNN demonstrates high stability and generally outperforms a single algorithm in all scenarios. Additionally, to ascertain the statistical significance of the hybrid algorithm’s performance, we conducted p-value calculations using the t-test method on the experimental results of the hybrid algorithm under different latent factors, as presented in Table 9:

**Table 9:** T-test experimental data table of the hybrid algorithm.

$\alpha$=0.05		Number of latent factors				
Algorithm	Metrics	16	32	48	64	80
UNN+ALS	p-F1	0.05130	0.03408	0.00318	0.04510	0.04142
	p-HR	0.02446	0.01143	0.06578	0.09764	0.08294
	p-NDCG	0.05527	0.02924	0.02163	0.03533	0.00194
UNN+MLP	p-F1	0.03780	0.00220	0.00936	0.03803	0.03191
	p-HR	0.09274	0.02791	0.07092	0.03641	0.01979
	p-NDCG	0.04564	0.07432	0.01954	0.03846	0.00523
UNN+NCF	p-F1	0.04806	0.01599	0.05156	0.01051	0.03600
	p-HR	0.00433	0.05895	0.03617	0.07926	0.00475
	p-NDCG	0.05142	0.05312	0.01036	0.05947	0.03358

Based on the analysis of the above experimental data, the hybrid recommendation algorithm we proposed combines the UNN model and collaborative filtering method. By adjusting the similarity threshold, it effectively optimizes the user-item interaction matrix and mitigates matrix sparsity to improve the quality of recommendations. Furthermore, t-test results indicate that in most cases, there is a 95% probability that the performance of the hybrid algorithm significantly differs from that of a single algorithm.

However, the effectiveness of this algorithm largely hinges on the optimization achieved by the UNN model on the user-item interaction matrix. Unfortunately, the UNN model in this paper relies on user similarity, which may lead to insufficient optimization of the user-item interaction matrix. Therefore, future research could explore more efficient and accurate algorithms to enhance the UNN model.

For instance, integrating graph neural networks (GNNs)^31^ could help capture implicit relationships between users, while combining multiple information sources such as user behavior data, social network information, and product content for feature fusion could improve the accuracy of user similarity calculations under sparse data conditions. This approach would effectively enhance the optimization effect of the UNN model on the user-item interaction matrix.

## Conclusion

Collaborative filtering remains a crucial area of research within recommender systems. This study introduces a novel approach focusing on optimizing the user-item interaction matrix using the user nearest neighbor (UNN) model, which refines the training matrix for collaborative filtering algorithms. Additionally, we integrate the UNN model with multiple recommendation algorithms to evaluate the effectiveness of our hybrid recommendation approach. Experimental results indicate that this strategy significantly reduces the negative effects of matrix sparsity on collaborative filtering algorithms. Furthermore, leveraging a distributed platform enables efficient processing of large-scale matrices, thereby enhancing model training efficiency.

The recommendation quality of the hybrid recommendation algorithm proposed in this paper heavily relies on the optimization effectiveness of the UNN model for the user-item interaction matrix. Moreover, the accuracy of user similarity measurement is crucial for the efficacy of the UNN model. In scenarios where user and item interactions are sparse, the precision of user similarity measurement may decrease, thereby affecting the overall recommendation quality of the algorithm. Furthermore, when dealing with larger datasets, enhancing the hybrid recommendation algorithm’s capability to mitigate data sparsity becomes imperative. Future research efforts could focus on improving user similarity measurement accuracy and enhancing the algorithm’s scalability to address these challenges effectively.

## Data Availability

The datasets used and/or analyzed during the current study available from the corresponding author on reasonable request.

## References

[1] Adomavicius, G. & Tuzhilin, A. Toward the next generation of recommender systems: A survey of the state-of-the-art and possible extensions. *IEEE Trans. Knowl. Data Eng.* **17**, 734–749 (2005). 10.1109/TKDE.2005.99

[2] Ricci, F., Rokach, L. & Shapira, B. *Recommender Systems Handbook* **1–35**, 1–35 (2010).

[3] Kumar, B. Approaches, issues and challenges in recommender systems: A systematic review. *Indian J. Sci. Technol.* **9**, 1–12 (2016).

[4] Zhang, Q., Lu, J. & Jin, Y. Artificial intelligence in recommender systems. *Complex Intell. Syst.* **7**, 439–57 (2020). 10.1007/s40747-020-00212-w

[5] Feng, Y. Enhancing e-commerce recommendation systems through approach of buyer’s self-construal: Necessity, theoretical ground, synthesis of a six-step model, and research agenda. *Front. Artif. Intell.* **6**, 1167735 (2023). 37293239 10.3389/frai.2023.1167735 PMC10244742

[6] Dixit, V. & Gupta, S. *Personalized Recommender Agent for E-Commerce Products Based on Data Mining Techniques: Proceedings of ISTA* Vol. 2018, 77–90 (Springer, 2020).

[7] Sarwar, B., Karypis, G., Konstan, J. & Riedl, J. Item-based collaborative filtering recommendation algorithms. In *Proceedings of the 10th International Conference on World Wide Web* (ed. Sarwar, B.) 285–295 (Association for Computing Machinery, 2001).

[8] Linden, G., Smith, B. & York, J. Amazon.com recommendations: Item-to-item collaborative filtering. *IEEE Internet Comput.* **7**, 76–80 (2003). 10.1109/MIC.2003.1167344

[9] Chen, R. *et al.* A survey of collaborative filtering-based recommender systems: From traditional methods to hybrid methods based on social networks. *IEEE Access* **6**, 64301–64320 (2018). 10.1109/ACCESS.2018.2877208

[10] Cacheda, F., Carneiro, V., Fernández, D. & Formoso, V. Comparison of collaborative filtering algorithms: Limitations of current techniques and proposals for scalable, high-performance recommender systems. *ACM Trans. Web* **5**, 1–33 (2011). 10.1145/1921591.1921593

[11] Lee, J.-S. & Olafsson, S. Two-way cooperative prediction for collaborative filtering recommendations. *Expert Syst. Appl.* **36**, 5353–5361 (2009). 10.1016/j.eswa.2008.06.106

[12] Liu, H., Hu, Z., Mian, A., Tian, H. & Zhu, X. A new user similarity model to improve the accuracy of collaborative filtering. *Know.-Based Syst.* **56**, 156–166 (2014). 10.1016/j.knosys.2013.11.006

[13] Kim, S.-C., Sung, K.-J., Park, C.-S. & Kim, S. Improvement of collaborative filtering using rating normalization. *Multim. Tools Appl.* **75**, 4957–68 (2013). 10.1007/s11042-013-1814-0

[14] Koren, Y., Bell, R. & Volinsky, C. Matrix factorization techniques for recommender systems. *Computer* **42**, 30–37 (2009). 10.1109/MC.2009.263

[15] LeCun, Y., Bengio, Y. & Hinton, G. Deep learning. *Nature* **521**, 436–44 (2015). 26017442 10.1038/nature14539

[16] He, X. *et al.* Neural collaborative filtering. 173–182 (International World Wide Web Conferences Steering Committee, 2017).

[17] Kumar, P. & Thakur, R. Recommendation system techniques and related issues: A survey. *Int. J. Inf. Technol.* **10**, 495–501 (2018).

[18] Burke, R. Hybrid recommender systems: Survey and experiments. *User Modeling and User-Adapted Interaction* (2002).

[19] Jitao, F. *Research and Application of Recommendation System Based on Spark Platform* (Dalian Maritime University, 2018).

[20] Congcui, J. & Qiaoling, C. Construction and application of real-time recommendation system for e-commerce based on spark platform. *Electron. Commer.* 65–66+94 (2020).

[21] Lianyue, Z. *Research and Implementation of Movie Recommendation System Based on Flink* (University of Electronic Science and Technology, 2020).

[22] Zhang, T., Zhang, Y., Zhang, G., Xue, L. & Wang, J. De privacy encryption and extraction model of smart grid data based on spark streaming. *J. Intell. Fuzzy Syst.* **43**, 6821–6830 (2022). 10.3233/JIFS-221185

[23] Omar, H. & Jumaa, A. Big data analysis using apache spark mllib and hadoop hdfs with scala and java. *Kurdistan J. Appl. Res.* **4**, 7–14 (2019). 10.24017/science.2019.1.2

[24] Ramesh, D. & Arora, N. Spark’s graphx-based link prediction for social communication using triangle counting. *Soc. Netw. Anal. Min.* **9**, 28 (2019). 10.1007/s13278-019-0573-y

[25] R, M. T., Kumar, V. V. & Lim, S.-J. Uscotc: Improved collaborative filtering (cfl) recommendation methodology using user confidence, time context with impact factors for performance enhancement. *PLOS ONE* **18**, e0282904 (2023). 10.1371/journal.pone.0282904 PMC10016635 36921014

[26] Isinkaye, F., Folajimi, Y. & Ojokoh, B. Recommendation systems: Principles, methods and evaluation. *Egypt. Inform. J.* **16**, 261–273 (2015). 10.1016/j.eij.2015.06.005

[27] Lin, L., Peipei, W., Peng, G. & Qing, X. Distributed singular value decomposition recommendation algorithm based on lu decomposition and alternating least squares approach. *Pattern Recogn. Artif. Intell.* **33**, 32–40 (2020).

[28] Gupta, S. & Dixit, V. Scalable online product recommendation engine based on implicit feature extraction domain. *J. Intell. Fuzzy Syst.* **34**, 1503–1510 (2018). 10.3233/JIFS-169445

[29] Kim, Y. & Yum, B.-J. Recommender system based on click stream data using association rule mining. *Expert Syst. Appl.* **38**, 13320–13327 (2011). 10.1016/j.eswa.2011.04.154

[30] Kumar, S., Singh, J., Jain, V. & Marahatta, A. A deep ranking weighted multihashing recommender system for item recommendation. *Computat. Intell. Neurosci.* **2022**, 7393553 (2022). 10.1155/2022/7393553 PMC9576357 36262607

[31] Xv, G. *et al.**E-commerce Search Via Content Collaborative Graph Neural Network* 2885–2897 (Association for Computing Machinery, 2023).

